# The association between higher nurse staffing standards in the fee schedules and the geographic distribution of hospital nurses: A cross-sectional study using nationwide administrative data

**DOI:** 10.1186/s12912-017-0219-1

**Published:** 2017-05-23

**Authors:** Noriko Morioka, Jun Tomio, Toshikazu Seto, Yasuki Kobayashi

**Affiliations:** 1 Department of Public Health, Graduate School of Medicine, The University of Tokyo, 7-3-1 Hongo, Bunkyo-ku, Tokyo, 113-0033 Japan; 2 Department of Gerontological Nursing and Care System Development, Graduate School of Health Care Science, Tokyo Medical and Dental University, 1-5-45 Yushima, Bunkyo-ku, Tokyo, Japan; 3 Center for Spatial Information Science, The University of Tokyo, 4-6-1 Komaba, Meguro-ku, Tokyo, 153-8505 Japan

**Keywords:** Geographic distribution of nurses, Health resources, Health services geographic accessibility, Nurse staffing, Nurse workforce, Patient-to-nurse ratio

## Abstract

**Background:**

In Japan, the revision of the fee schedules in 2006 introduced a new category of general care ward for more advanced care, with a higher staffing standard, a patient-to-nurse ratio of 7:1. Previous studies have suggested that these changes worsened inequalities in the geographic distribution of nurses, but there have been few quantitative studies evaluating this effect. This study aimed to investigate the association between the distribution of 7:1 beds and the geographic distribution of hospital nursing staffs.

**Methods:**

We conducted a secondary data analysis of hospital reimbursement reports in 2012 in Japan. The study units were secondary medical areas (SMAs) in Japan, which are roughly comparable to hospital service areas in the United States. The outcome variable was the nurse density per 100,000 population in each SMA. The 7:1 bed density per 100,000 population was the main independent variable. To investigate the association between the nurse density and 7:1 bed density, adjusting for other variables, we applied a multiple linear regression model, with nurse density as an outcome variable, and the bed densities by functional category of inpatient ward as independent variables, adding other variables related to socio-economic status and nurse workforce. To investigate whether 7:1 bed density made the largest contribution to the nurse density, compared to other bed densities, we estimated the standardized regression coefficients.

**Results:**

There were 344 SMAs in the study period, of which 343 were used because of data availability. There were approximately 553,600 full time equivalent nurses working in inpatient wards in hospitals. The mean (standard deviation) of the full time equivalent nurse density was 426.4 (147.5) and for 7:1 bed density, the figures were 271.9 (185.9). The 7:1 bed density ranged from 0.0 to 1,295.5. After adjusting for the possible confounders, there were more hospital nurses in the areas with higher densities of 7:1 beds (standardized regression coefficient 0.62, 95% confidence interval 0.56–0.68).

**Conclusion:**

We found that the 7:1 nurse staffing standard made the largest contribution to the geographic distribution of hospital nurses, adjusted for socio-economic status and nurse workforce-related factors.

**Electronic supplementary material:**

The online version of this article (doi:10.1186/s12912-017-0219-1) contains supplementary material, which is available to authorized users.

## Background

There is a growing body of evidence that nurse staffing levels make a difference to patient outcomes such as mortality, adverse events and length of hospital stay [1–5] . Mandated minimum nurse staffing ratios have been introduced in several states and countries to improve nurse staffing ratios and therefore patient outcomes [6]. In California, USA, evaluation of the effect of mandated minimum nurse staffing ratios on patient outcomes showed mixed results, but implementation of the mandate achieved the primary policy aim to improve nurse staffing in hospitals [7–13].

In Japan, the fee schedules set nurse staffing standards for each functional category of inpatient ward [14]. Reimbursement is higher for wards with higher nurse staffing ratios, which care for acute and/or more severe patients. Revision of the fee schedules in 2006 changed the nurse staffing indicator from patient-to-employed nurse ratios to patient-to-nurse ratios per shift, and introduced a new category of general care ward with a higher nurse staffing standard of a 7:1 patient-to-nurse ratio, to improve nurse staffing and quality of care. Before this revision, the highest nurse staffing standard was a 2:1 patient-to-employed nurse ratio, which equated to approximately a 10:1 patient-to-nurse ratio per shift [15]. Introduction of the 7:1 ratio has succeeded in increasing the number of nursing staff and reducing the average length of hospital stay [16, 17].

Introduction of the 7:1 ratio has succeeded in increasing the number of nursing staff and reducing the average length of hospital stay [16, 17]. Some experts’ opinions and case reports, however, suggested that inequitable distribution of beds with a 7:1 ratio has made the geographic distribution of nurses worse [18–21]. Large or well-funded hospitals have been able to employ more nurses to meet the 7:1 ratio requirements, and therefore increase their reimbursement levels. However, small or less well-funded hospitals, mostly in rural areas, have been unable to do this. In other words, the financial incentives to increase nurse staffing and to improve quality of care might lead to partial rather than total optimization of hospital services in Japan. No quantitative studies, however, have examined the geographic distribution of beds with a 7:1 ratio and their association with the geographic distribution of hospital nurses.

The number of nursing and midwifery personnel per 1,000 population in Japan was 11.489 and the 12th highest in the world [22], but the unequal distribution and relative shortage of nurses in some areas in Japan has been a concern [23]. To achieve the optimal geographic distribution of nurses, it is necessary to use nationwide administrative data to investigate whether the introduction of the higher nurse staffing ratio in the fee schedule could be associated with an unequal distribution of nurses. This study therefore aimed 1) to describe the nationwide geographic distribution of beds with a 7:1 ratio; and 2) to quantify the degree of the association between the geographic distribution of 7:1 beds and of hospital nursing staff.

## Methods

### Study design

We conducted a cross-sectional study using nationwide data from hospital reimbursement reports for the health insurance system. From the hospital reports, we obtained data on nurse staffing for all the hospitals in operation in 2012, disclosed by the Ministry of Health, Labour, and Welfare. No individual data were used, only aggregate data disclosed by the Ministry, based on the Act on Access to Information Held by Administrative Organs [24]. The study was consistent with the Declaration of Helsinki.

### Study setting and areal unit of analysis

We used secondary medical areas (SMAs) as areal units of analysis. The Japanese administrative system is organized into three tiers of administration: national, prefectural, and municipal. Japan had 47 prefectures and 1,719 municipalities in January 2013 [25]. SMAs are considered independent administrative areas from a health service perspective, and typically include several municipalities. Each prefecture defined the medical area boundaries taking into account medical resources, transportation, and geography. In each SMA, the total number of hospital beds offering general inpatient care is regulated based on the Medical Care Act [26]. The boundaries from 2013 were used in this study, giving 344 areas in total, of which 343 were used for the analyses. The average population of each area was 230,000 people, of whom 25.9%, on average, were aged 65 or older [27].

### The nursing system in Japan

In Japan, nurse and associate nurse qualifications are set out by the Act on Public Health Nurses, Midwives, and Nurses [28]. Nurses require a national license, and associate nurses are licensed by the prefectural governors, but both licenses are effective anywhere in Japan. Nurses have to study for at least 3 years at nursing school or university, and associate nurses for 2 years. While the practical training and educational achievements differ, associate nurses are permitted to provide nursing services under the direction of a physician, dentist or nurse. Nursing aides are not required to be qualified and assist nurses in providing personal care of patients under nurses’ supervision, and housekeeping work, such as washing laundry, cleaning up, and clerical tasks in hospitals [29].

### Nurse staffing standards in Japan

Japan introduced nurse staffing standards in the Medical Care Act and the fee schedules to maintain adequate nurse staffing ratios in hospitals. The Medical Care Act sets the standards for minimum inpatient-to-employed nurse ratios for five functional categories of beds: 3:1 for general care and infectious disease beds; 4:1 for long-term care and tuberculosis beds; and either 3:1 or 4:1 for psychiatric beds. For example, if there were 60 patients in a ward of general care beds, more than 20 full-time equivalent (FTE) nurses should be employed there.

The fee schedule also sets the standard for nurse staffing ratios for each functional category of inpatient ward. Currently there are more than 20 of these categories. In principle, the fee schedules differ by functional category, and reimbursement is usually higher for wards with higher nurse staffing ratios, as these take more acute and seriously ill patients. Hospitals can apply to have any functional categories of inpatient ward, regardless of their size, ownership, function, and location, provided they meet certain criteria, such as minimum nurse staffing ratios, average length of hospital stay and the required level on the nursing necessity scale based on a patient classification index, adjusting for patient case mix.

### Variables and data sources

#### Hospital nurse density

As variables for geographic distribution of nursing staffs, we used the densities of nursing staffs (nurse, associate nurse, and nursing aid), calculated as the number of nursing staffs in inpatient wards in hospitals per 100,000 population in each SMA. The nurse density is often used to assess the geographical distribution of health professionals [30]. The numbers of nursing staffs were obtained from full-time equivalent (FTE) hospital employees. The number of FTE hospital employees for part-time personnel was calculated by dividing full-time worker’s working time by actual working time. Nurses included midwives and public health nurses who were employed by hospitals. These data were obtained from reports about basic hospitalization charges in 2012 [31]. We collected these reports for all 8,479 hospitals in Japan by requesting disclosure of administrative documents from the Regional Bureaus of the Ministry of Health, Labour, and Welfare on October 15, 2013, based on the Act on Access to Information Held by Administrative Organs [24]. In July each year, all hospitals have to submit routine reports to the relevant Regional Bureau office, for reimbursement from the public health insurance system, based on actual data during June. The reports contain information about the numbers of hospital beds and nursing staff, including hospital address (municipality), ownership, and type of units and/or wards. The population of each area was calculated using the population of municipalities, obtained from the Surveys of Population, Population Change and the Number of Households, and based on the Basic Resident Registration on March 31, 2012 [32].

#### Hospital bed densities by functional categories in fee schedule

Since April 2012, there have been four types of general ward, with inpatient-to-nurse ratios of 7:1; 10:1; 13:1 and 15:1. Although the inpatient-to-nurse ratio includes both nurses and associate nurses, there are lower limits set on the proportion of nurses (Additional file 1). In long-term care wards, there are also standards for nursing aides.

As the variables for geographic distribution of hospital beds of each functional category, we used the densities of hospital beds, calculated as the number of hospital bed per 100,000 population in each SMA: general care wards with inpatient-to-nurse ratio of 7:1, 10:1, 13:1, 15:1; intensive care units; LTC wards; and psychiatric wards. The intensive care unit beds included beds in emergency departments, neo-natal intensive care units, high dependency units, stroke care units, pediatric intensive care units, and maternal-fetal intensive care units, because those units represent the supply of tertiary emergency medical services. The number of beds at each hospital in 2012 was obtained from reports about basic hospitalization charges [31]. One area was excluded in the data-cleaning process as the number of 7:1 beds exceeded the total number of beds.

#### Socio-economic status and nurse workforce variables

As socio-economic variables of geographical areas [20, 21, 33], we used population density, per capita income and unemployment rate in 2015 [34]. We also used the standard number of beds to represent the age- and sex-adjusted risk for admission in each area. Each prefecture defines the standard number of beds per SMA, considering the demographic structure of the area [35]. For nurse workforce variables [36], we used nurses’ average annual wage, the number of graduates from nursing schools per 100,000 population, and nurse turnover rate. Data at prefecture level were substituted because of data availability. Nurses’ average annual wage was obtained from the 2012 Basic Survey on Wage Structure [37]. The number of graduates was the number of students who graduated from all kinds of nursing schools, including university and college, in the prefecture in March 2011 [38]. Turnover rate in 2011 was obtained from the Nursing Staff Supply and Demand Situation Survey in Hospitals in 2013 [39].

#### Statistical analysis

We described the mean and standard deviation (SD) of the nursing density, bed densities and other variables. We showed the geographic distribution of the 7:1 bed density on a map using QGIS version 2.14. All spatial sources were obtained from the National Land Numerical Information download service [40].

To investigate the association between each category of nursing staffs densities and 7:1 bed density, we performed multivariate linear regression analysis adjusting for socio-economic status and nurse workforce variables as covariates. We used cluster robust standard errors at the prefecture level to take into account the correlation of residuals within prefectures. To investigate whether the 7:1 bed density had the largest association with the nurse density, compared to other bed densities, we estimated the standardized regression coefficients. We used population density quartiles as dummy variables, with the first quartile as a reference. All numeric independent variables were centered to the overall mean. We checked on our data meet the assumptions of multiple linear regression using geographic methods and numerical test such as variance inflation factors to check for multicollinearity. A two-tailed *p*-value of < 0.05 was considered statistically significant. All analyses were performed with Stata version 13.1 (StataCorp. College Station, TX, USA).

## Results

In 2012, the numbers of FTE nurses, associate nurses, and nursing aids who working in inpatient wards in hospitals were approximately 553,600, 104,700, and 185,400, respectively. There were 1.46 million hospital beds in total, of which 413,300 were 7:1 beds, the largest functional category (Table 1).

**Table 1:** Total number of nursing staff and hospital beds in 343 secondary medical areas Japan

Variables	Total number (×1,000)
Number of FTE nurses in inpatient wards	553.6
Wards with a 7:1 patient-to-nurse ratio	241.8
Wards with a 10:1 patient-to-nurse ratio	83.8
Wards with a 13:1 patient-to-nurse ratio	8.0
Wards with a 15:1 patient-to-nurse ratio	12.1
Intensive care units^a^	47.0
Long-term care wards	30.6
Psychiatric wards	47.1
Other wards	83.2
Number of FTE associate nurses in inpatient wards	104.7
Wards with a 7:1 patient-to-nurse ratio	7.7
Wards with a 10:1 patient-to-nurse ratio	11.3
Wards with a 13:1 patient-to-nurse ratio	2.6
Wards with a 15:1 patient-to-nurse ratio	7.1
Intensive care units^a^	0.2
Long-term care wards	26.9
Psychiatric wards	29.3
Other wards	19.7
Number of FTE nursign aids in inpatient wards	185.4
Wards with a 7:1 patient-to-nurse ratio	30.2
Wards with a 10:1 patient-to-nurse ratio	19.5
Wards with a 13:1 patient-to-nurse ratio	3.5
Wards with a 15:1 patient-to-nurse ratio	7.5
Intensive care units^a^	1.4
Long-term care wards	55.2
Psychiatric wards	2.6
Other wards	65.4
Number of hospital beds	1454.4
Beds with a 7:1 patient-to-nurse ratio	413.3
Beds with a 10:1 patient-to-nurse ratio	207.1
Beds with a 13:1 patient: nurse ratio	26.8
Beds with a 15:1 patient-to-nurse ratio	53.2
Intensive care unit beds^a^	21.4
Long-term care beds	212.8
Psychiatric ward beds	260.6
Others^b^	259.2

*FTE* full time equivalent

^a^Intensive care units include emergency departments, high dependency units, stroke care units, neonatal and pediatric intensive care units, and maternal-fetal intensive care units

^b^Others includes infectious disease, sub-acute, and rehabilitation beds

Table 2 shows the densities per 100,000 population of hospital nurses, and 7:1, 10:1, 13:1, 15:1, intensive care, LTC, and psychiatric beds. The mean (standard deviation, SD) of the densities of nurse, associate nurse and nursing aid were 426.4 (147.5), 103.2(61.9), and 161.9 (72.2), respectively. The mean (SD) of 7:1 bed density was 271.9 (185.9).

**Table 2:** Characteristics of 343 secondary medical areas in Japan

*n* = 343		
	Mean	SD
Outcome variable		
Nurses per 100,000 populations	426.4	147.5
Associate nurses per 100,000 populations	103.2	61.9
Nursing aids per 100,000 populations	161.9	72.2
Independent variables		
Hospital bed densities per 100,000 population		
Beds with a 7:1 patient-to-nurse ratio	271.9	185.9
Beds with a 10:1 patient-to-nurse ratio	231.3	188.9
Beds with a 13:1 patient-to-nurse ratio	24.2	41.8
Beds with a 15:1 patient-to-nurse ratio	49.3	55.8
Intensive care unit beds^a^	12.0	13.5
Long-term care beds	205.7	141.5
Psychiatric ward beds	220.2	168.4
Socioeconomic variables		
Population density (person/km^2^)	1692.6	2674.5
Unemployment rate (%)	6.4	1.4
Per capita income (100 thousand yen)	28.5	4.5
Standard number of beds in Prefectural Medical Care Plans (thousand beds)	3.1	3.3
Nurse workforce-related variables		
Average annual wage of nurses (100,000 yen)^b,c^	46.4	3.2
The number of graduates from nursing schools per 100,000 population^c^	43.4	12.4
The number of graduates from associate nursing schools per 100,000 population^c^	10.0	7.5
Turnover rate (%)^c^	10.1	2.2

^a^Intensive care units include emergency departments, high dependency units, stroke care units, neonatal and pediatric intensive care units, and maternal-fetal intensive care units

^b^1 US$ = 109 yen as of June 3, 2016

^c^The data at each prefecture level were substituted

**Fig. 1:**
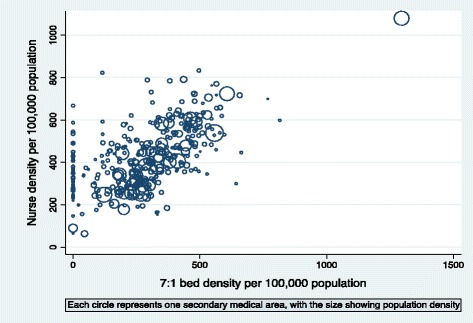
Association between nurse density and 7:1 bed density in 343 secondary medical areas in Japan

**Fig. 2:**
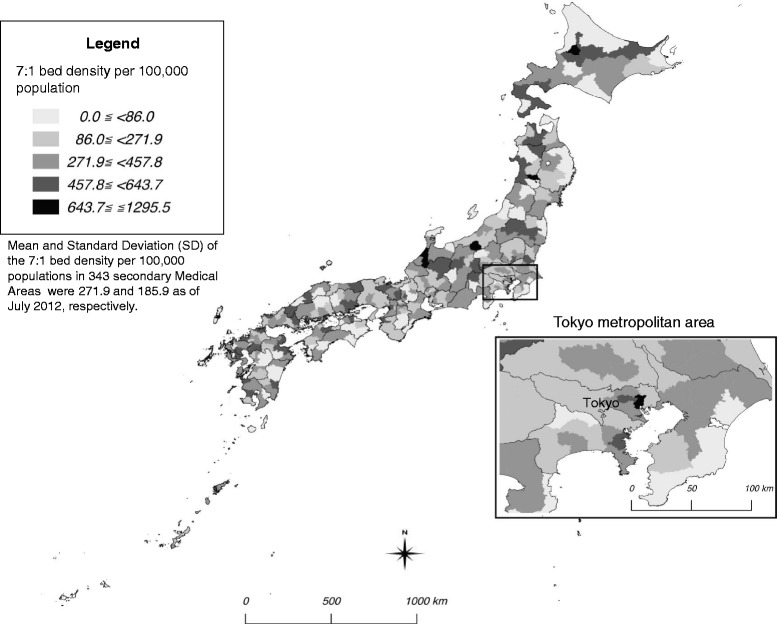
Geographic distribution of the 7:1 bed density in 343 secondary medical areas in Japan. Author created the map based on spatial vector data of secondary medical areas obtained from National Land Numerical Information download service (http://nlftp.mlit.go.jp/ksj-e/index.html), Ministry of Land, Infrastructure, Transport and Tourism (Accessed 14.09.19)

The 7:1 bed density ranged from 0.0 to 1,295.5 with approximately 17% of the 343 SMAs, mostly in areas with lower population densities, having no 7:1 beds. A higher 7:1 bed density was associated with a higher nurse density (Fig. 1). The 7:1 bed density varied within prefectures (Fig. 2).

After adjusting for the variables related to socio-economic status and nurse workforce, higher 7:1 bed density was significantly associated with higher nurse density (regression coefficient (*β*) 0.49, 95% confidence interval (CI) 0.41–0.57), associate nurse density (*β* 0.05, 95% CI 0.01–0.10), and nursing aid density (*β* 0.09, 95% CI 0.05–0.13) (Table 3). These indicates that, in an average size SMA with 200 thousand population, about two, 20 and 10 extra 7:1 beds were associated with one extra nurse, associate nurse, and nursing aid, respectively.

**Table 3:** Result of linear regression analysis for the number of nursing staff per 100,000 population

	Nurse density						Associate nurse density						Nursing aid density					
	Non-standardized^d^			Standardized			Non-standardized^d^			Standardized			Non-standardized^d^			Standardized		
	β	95% CI		β	95% CI		β	95% CI		β	95% CI		β	95% CI		β	95% CI	
Hospital bed densities per 100,000 population																		
Beds with a 7:1 patient-to-nurse ratio	0.49	0.41	0.57^***^	0.62	0.56	0.68^***^	0.05	0.01	0.10^*^	0.16	0.09	0.23^***^	0.09	0.05	0.13^***^	0.24	0.17	0.30^***^
Beds with a 10:1 patient-to-nurse ratio	0.36	0.29	0.43^***^	0.46	0.39	0.53^***^	0.05	0.03	0.08^***^	0.15	0.09	0.22^***^	0.10	0.06	0.13^***^	0.25	0.19	0.31^***^
Beds with a 13:1 patient-to-nurse ratio	0.24	0.05	0.44^*^	0.07	0.02	0.12^*^	0.02	−0.05	0.09	0.01	−0.04	0.06	0.06	−0.03	0.14	0.03	−0.01	0.08
Beds with a 15:1 patient-to-nurse ratio	0.33	0.19	0.47^***^	0.12	0.07	0.18^***^	0.22	0.15	0.28^***^	0.20	0.14	0.25^***^	0.12	0.03	0.21^*^	0.09	0.04	0.14^***^
Intensive care unit beds^a^	3.27	2.34	4.21^***^	0.30	0.24	0.36^***^	−0.06	−0.42	0.30	−0.01	−0.07	0.05	0.29	0.03	0.55^*^	0.05	0.00	0.11
Long-term care ward beds	0.18	0.09	0.27^***^	0.17	0.11	0.24^***^	0.18	0.13	0.23^***^	0.42	0.36	0.47^***^	0.29	0.25	0.32^***^	0.56	0.51	0.61^***^
Psychiatric ward beds	0.17	0.11	0.22^***^	0.19	0.13	0.25^***^	0.11	0.08	0.14^***^	0.30	0.24	0.36^***^	0.15	0.13	0.17^***^	0.34	0.29	0.40^***^
Socioecomic variables																		
Population density (refefence: 1st quartile)																		
2nd quartile	31.78	1.35	62.21^*^	0.09	0.03	0.16^*^	−18.78	−32.79	−4.77*	−0.13	−0.19	−0.07^***^	−3.95	−19.03	11.13	−0.02	−0.08	0.03
3rd quartile	10.12	−18.94	39.18	0.03	−0.05	0.10	−14.01	−27.69	−0.32*	−0.10	−0.17	−0.03**	−9.83	−27.30	7.63	−0.06	−0.13	0.01
4th quartile	24.32	−12.67	61.32	0.07	−0.04	0.18	−17.39	−32.75	−2.04*	−0.12	−0.23	−0.02*	−6.79	−31.05	17.48	−0.04	−0.14	0.06
Unemployment rate (%)	−2.12	−8.29	4.04	−0.02	−0.08	0.04	2.71	−0.78	6.20	0.06	0.01	0.12*	2.74	−0.43	5.90	0.05	0.00	0.11*
Per capita income (100,000 yen/person)	3.08	−0.08	6.24	0.09	0.00	0.19	−1.76	−2.72	−0.80**	−0.13	−0.22	−0.04**	−1.56	−2.72	−0.41**	−0.10	−0.18	−0.01*
Standard number of beds in Prefectural Medical Care Plans (1,000 beds)	4.16	0.86	7.46^*^	0.09	0.02	0.17^*^	−1.23	−2.12	−0.33**	−0.07	−0.13	0.00	0.17	−0.99	1.33	0.01	−0.06	0.07
Nurse workforce related variables																		
Average annual wage of nurses (100,000 yen)^b,c^	−8.53	−12.66	−4.41^***^	−0.18	−0.26	−0.11^***^	−1.18	−2.99	0.62	−0.06	−0.13	0.01	−1.22	−3.16	0.71	−0.05	−0.12	0.02
The number of graduates from nursing schools per 100,000 population^c^	−2.10	−16.33	12.12	−0.01	−0.07	0.05							0.00	−0.34	0.34	0.00	−0.06	0.06
The number of graduates from associate nursing schools per 100,000 population^c^							1.32	0.59	2.04**	0.16	0.10	0.22***						
Turnover rate^c^	−7.82	−13.25	−2.40**	−0.12	−0.19	−0.05**	5.26	2.59	7.94***	0.19	0.12	0.25***	5.78	2.86	8.69***	0.176	0.11	0.24***
Adj R-squared	0.76						0.79						0.82					

^*^ *p <* 0.05, ^**^ *p <* 0.01, ^***^ *p <* 0.001

^a^Intensive care units include emergency departments, high dependency units, stroke care units, neonatal and pediatric intensive care units, and maternal-fetal intensive care units

^b^1 US$ = 109 yen as of June 3, 2016

^c^The data at prefecture level were substituted

^d^With clusters robust standard errors at the prefecture level

The standardized regression coefficients of nurse, associate nurse, and nursing aid were the largest: the 7:1 density (standardized β 0.62, 95% CI 0.56–0.68); the LTC bed density (standardized β 0.42, 95% CI 0.36–0.47); and the LTC bed density (standardized β 0.56, 95% CI 0.51–0.61), respectively.

Higher nurse densities were also significantly associated with population density (2nd quartile vs. 1st quartile) (*β* 31.78, 95% CI 1.35–62.21), standard number of beds specified in prefectural medical care plans (*β* 4.16, 95% CI 0.86–7.46), lower annual nursing wages (*β* −8.53, 95% CI −12.66– − 4.41, and lower nurse turnover rate (*β* −7.82, 95% CI −13.25– − 2.4).

## Discussion

The hospital bed density with 7:1 patient-to-nurse ratio, i.e. the highest nurse allocation with highest reimbursement for general care, widely varied across the SMAs, from 0 to over the mean plus 4SD. We also found that the geographic distribution of hospital nurses was more closely associated with the geographic distribution of beds with a 7:1 patient-to-nurse ratio than with other bed densities, and adjusted for the socio-economic status and nurse workforce-related factors.

Originally, 7:1 beds for general care were supposed to be evenly distributed across SMAs, as those are considered independent administrative areas by the health service. Our finding, however, suggests that the distribution of 7:1 beds is not equal. We guess one of the reasons is that there is no formal cap on the total number of 7:1 beds in each SMA, and hospitals can provide 7:1 beds regardless of socio-economic status and/or other medical care provision in their areas. There is an incentive to have beds with higher nursing ratios, because the reimbursement in the fee schedules is higher for these beds [17]. Hospitals that are financially well-off and/or located in densely-populated areas have therefore been keen to recruit nurses. Moreover, providing 7:1 beds itself attracts nurses due to nurses willing to work at hospitals with lower burden [41]. As a result, the introduction of the 7:1 patient-to-nurse ratio has improved the quality of nursing care by increasing levels of nursing staff and shortening the average length of stay at hospitals, but mostly in urban areas [16, 17]. In total, however, 17% of the 343 SMAs, mostly in rural and/or remote areas, had no hospitals with even one 7:1 bed, and suffered from continuous nurse shortages. In other words, such financial incentives might lead to partial optimization rather than total optimization for delivery of hospital services in Japan.

Our finding suggested that higher staffing standards are associated with higher nurse densities, and especially that the 7:1 patient-to-nurse ratios, the highest level, had the greatest association with increased nurse density. This is consistent with previous case reports, which pointed out that mandatory minimum nurse staffing ratios in the fee schedule increased demand for nurses at the hospital level [7, 11–13]. We found a similar association between higher staffing ratios and higher nursing staff density among both associate nurses and nursing aides. Long-term care beds, which have a nurse skill mix standard that is 20% lower than other categories, and an additional nursing aide staffing standard (Additional file 1), had the greatest association with higher densities of associate nurses and nursing aides. Those findings are plausible because the minimum number of employed nursing staff depends on the nurse staffing standards, both patient-to-nursing staff ratios per shift and the proportion of nurses to total nursing staff.

These findings suggested that the unequal distribution of 7:1 beds is one of determinants of the unequal distribution of nurses. This is consistent with previous case reports suggesting that inequitable distribution of beds with high nurse staffing standards had a negative association with the geographic distribution of nurses [18, 19]. To achieve the optimal distribution of nurses, it is necessary to improve the distribution of 7:1 beds in SMAs, adjusting for socioeconomic status and healthcare needs. Putting caps on the number of hospital beds in each functional category in each SMA could be helpful. The Japanese Ministry of Health, Labour, and Welfare introduced a law in 2015 about the development of the vision for local medical care in each prefecture, to promote differentiation of hospital bed function in each SMA. This also put caps on the number of hospital beds for each functional category: advanced acute, acute, sub-acute, and long-term care [42]. These policy changes are expected to improve the unequal distribution of both hospital beds and nursing staff.

In Japan, previous studies have shown that higher numbers of nurses tend to be concentrated in urban areas with a higher population density and higher average income and/or prefecture capitals [20, 21]. Our findings are similar, as nurse densities were likely to be higher in areas with higher average per capita income and/or higher population density, but the association between population density and nurse density was not statistically significant. Our findings also show that nursing wages and nurse turnover rate were associated with nurse density. The negative association between nursing wages and nurse density might be because hospitals in areas with shortages of nurses would be more likely to increase wages to attract new staff. The negative association between nurse turnover rate and nurse density is similar to the finding of a previous study, in which nurses’ intention to leave had a negative association with nurse staffing levels at the hospital level [7].

This study had several limitations. First, we might have overestimated the number of nurses, because we used hospital-provided reports that are used for reimbursement from the health insurance system. Hospitals might overestimate the numbers of nurses to obtain more payment. However, false reporting, if discovered, results in the hospital having to refund the payment, which should prevent any deliberate falsification. The total number of FTE hospital nurses in this study was roughly the same as the figure given in the Report on Public Health Administration and Services on December 31, 2012 [43]. Second, SMAs in the same prefecture share tertiary emergency medical care resources, which are controlled by prefectural health policies. The nurse densities would be higher in those areas with emergency medical care centers with intensive care units and more 7:1 wards. We might therefore overestimate the degree of association between 7:1 bed density and nurse density across SMAs. However, we adjusted for this effect using the intensive care bed density as a proxy. Third, this study was cross-sectional, so cannot indicate any causal association between introduction of higher nurse staffing standards in the fee schedules, and the geographic distribution of nurses. However, it is implausible that the pre-existing unequal distribution of nurses might have caused the inequality in distribution of 7:1 beds [41].

Further investigation is needed to identify the healthcare system and/or financial incentives that would achieve the optimal distribution of nursing staff, adjusting for socioeconomic status and healthcare needs of each area.

## Conclusion

This study suggests that the revision of the fee schedules, to increase nurse staffing ratios, has the largest association with the geographic distribution of hospital nurses, adjusted for socio-economic status and nurse workforce-related factors. Ongoing examination of the distribution of nurses is needed following any changes in nurse staffing standards.

## Additional file

Additional file 1: The standards of inpatient-to-nurse ratios in the schedule of fees for inpatient wards in Japan (Extract). Additional file 1 shows the standards of inpatient-to-nurse ratios in the schedule of fees for inpatient wards in Japan as of July 2012. (XLSX 11 kb)

## Abbreviations

FTE: Full-time equivalent
LTC: Long-term care
SMA: Secondary medical area
